# Molecular evidence of recent hybridization between eastern and western populations of a whitefly species on cassava in the Democratic Republic of the Congo: A potential threat to the spread of cassava brown streak disease

**DOI:** 10.1371/journal.pone.0338200

**Published:** 2026-03-31

**Authors:** Olivier Likiti Kola, Christophe Simiand, Hadija Mussa Ally, Sharon L. van Brunschot, Justin S. Pita, John Colvin, Godefroid Monde Te Kazangba, Hélène Delatte

**Affiliations:** 1 Université de La Réunion, La Réunion, France; 2 CIRAD, UMR PVBMT, F-97410 St Pierre, La Réunion, France; 3 Laboratoire de Phytopathologie et Biotechnologies Végétales, Institut Facultaire des Sciences Agronomiques de Yangambi, Yangambi, Democratic Republic of the Congo; 4 Central and West African Virus Epidemiology (WAVE) for Food Security Program, Institut Facultaire des Sciences Agronomiques de Yangambi, Yangambi, Democratic Republic of the Congo; 5 Tanzania Agricultural Research Institute (TARI)–Ukiriguru Centre, Mwanza, Tanzania; 6 CSIRO Health & Biosecurity, Dutton Park, Queensland, Australia; 7 Natural Resources Institute, University of Greenwich, Chatham Maritime, Kent, United Kingdom; 8 UFR Biosciences, Université Félix Houphouet-Boigny (UFHB), Abidjan, Côte d’Ivoire; 9 The Central and West African Virus Epidemiology (WAVE) for Food Security Program, Pôle Scientifique et d’Innovation, Université Félix Houphouet-Boigny (UFHB), Abidjan, Côte d’ Ivoire; University of Florida Tropical Research and Education Center, UNITED STATES OF AMERICA

## Abstract

Cassava mosaic disease (CMD) and cassava brown streak disease (CBSD) are two viral diseases that threaten cassava production in the East and Central African countries. These diseases are spread by members of the cryptic species complex of the whitefly *Bemisia tabaci sensu lato,* and/or through the propagation of infected stem cuttings. This study aims to i) identify the *B. tabaci s.l.* species colonizing cassava in the north of the Democratic Republic of the Congo (DRC), ii) analyse their genetic diversity, and iii) examine how this diversity is geographically structured or influenced by invasions from neighbouring (eastern) countries with high CBSD prevalence. A comprehensive sampling survey was conducted across 43 sites from east to west in the DRC, spanning 1339 km. Both nuclear and mitochondrial markers were used to identify the species and study the genetic diversity and structuring of the populations. Three species of *B. tabaci s.l.* were found: *B. tabaci* SSA1-SG1 U SG2; *B. tabaci* SSA1-SG3, and *B.* *tabaci* SSA2 U SSA3. In the surveyed provinces, *B. tabaci* SSA1 SG1 U SG2 was the dominant species (94.91%). It was structured into two genetic clusters along the east-west transect, while *B. tabaci* SSA2 U SSA3 was restricted to the western provinces. The findings of this study confirm that *B. tabaci* SSA1-SG1 U SG2 is the most abundant and adapted species on cassava in the DRC. It is likely that this species is responsible for the spread of cassava virus diseases in the DRC. Furthermore, the results showed significant geographical structuring of *B. tabaci* SSA1-SG1 U SG2 populations, with potential movements of populations towards the west of the country. This highlights the increased risk of virus spread towards West Africa.

## Introduction

Cassava is one of the main staple food crop and a vital source of income for smallholder farmers across sub-Saharan Africa (SSA) [1]. It plays a key role in the food security of poor farmers in East and Central African countries [2]. In the Democratic Republic of the Congo (DRC), cassava is the most important and widely grown food crop, consumed in various forms by more than 70% of the population [3]. The tuberous roots are transformed into multiple products, while cassava leaves are consumed as a vegetable in all the provinces, varying by local preferences [4]. Other West African countries such as Benin, Côte d'Ivoire, Togo and Nigeria use cassava for human consumption in various artisanal products and as a source of raw material for a variety of industrial products such as starch, flour and ethanol [5]. Nigeria is the world's largest cassava producer with an estimated annual output of 63 million tonnes and its cassava transformation master plan is the most advanced in SSA [6].

Cassava mosaic disease (CMD) and cassava brown streak disease (CBSD) are the main constraint in cassava production in Sub-Saharan Africa. In the DRC, CMD is widespread in all provinces surveyed [7,8], although CBSD was initially reported in the eastern part of the country and is now spreading to the west [9]. The CMD is caused by a complex of DNA begomovirus species of the *Geminiviridae* family (genus *Begomovirus*). The CBSD is caused by two RNA viruses: *Ipomovirus brunusmanihotis* and *Ipomovirus manihotis* of the *Potyviridae* family (genus *Ipomovirus*) [10]. Both diseases are spread via the propagation of infected stem cuttings and are naturally vectored by members of the whitefly cryptic species complex *Bemisia tabaci s.l* with potentially different rates of transmission depending on the species [11,12].

The whitefly *B. tabaci s.l.* is recognised as one of the world’s most destructive agricultural pest [13]. It causes significant yield and quality losses directly by damage caused during feeding and the induction of physiological disorders, and indirectly by transmitting phytoviruses [14]. *B. tabaci s.l.* is recognized as a cryptic species complex [15]. Unravelling the taxonomy of these cryptic species was a serious challenge until the advancement of genetic and molecular biology tools, such as DNA sequencing [16]. Various molecular genetic tools have been used to differentiate phylogenetic species within *B. tabaci s.l.* [17], with barcoding studies using the mitochondrial cytochrome oxidase I (mtCOI) marker contributing significantly to this progress [18–20]. There are currently 44 putative cryptic biological species of *B. tabaci s.l.* recognised worldwide [21].

Across SSA, various studies have examined the *B. tabaci s.l.* species that colonize crops such as cassava, cotton, tomato, sweet potato and various other vegetable and weed species [22,23]. These studies have identified several putative *B. tabaci* SSA species within this species complex, named: *B. tabaci* SSA 1–7, and *B. tabaci* SSA 9–16, and reported their distribution in SSA countries [20]. Furthermore, genomic and biological evidence have recently identified and renamed three species within these putative species, occurring widely in SSA: *B. tabaci* SSA1-SG1 ∪ SG2, *B. tabaci* SSA2 ∪ SSA3 and *B. tabaci* SSA1-SG3 [24]. In addition to the *B. tabaci* Sub-Saharan African Sub-Group (SSA-SG) species, other species, including Mediterranean (MED), East Africa (EA), Indian Ocean (IO), and Middle East-Asia Minor 1 (MEAM1), have been shown to be present [18,25,26].

*B. tabaci* SSA1-SG1 ∪ SG2, and SSA2 ∪ SSA3 have been linked to the spread of cassava virus diseases in the Great Lakes region [27]. *B. tabaci* SSA2 ∪ SSA3 is the dominant species colonising cassava in Nigeria [28], and has also been reported in the Central African Republic [23], Cameroon, Benin, western DRC [27], and Uganda [26]. However, given the size of the DRC (2,345,000 km²), there is limited information on the genetic diversity and geographical distribution of the species of the *B. tabaci* complex [29], that could particularly contribute to the spread of CBSD on cassava toward West Africa. In the DRC, CBSD was first reported in 2012 in the eastern part of the country [30], and is now spreading towards the western region [9]. Therefore, this study aims to contribute to a better understanding of i) *B. tabaci s.l.* species diversity, and ii) its distribution on cassava, iii) the population structure within the species, in northern DRC, and iv) if a signal of population movement from the East (areas where outbreaks of CBSD have been detected) of the country could be detected.

## Materials and methods

### Whitefly sampling

**Fig 1:**
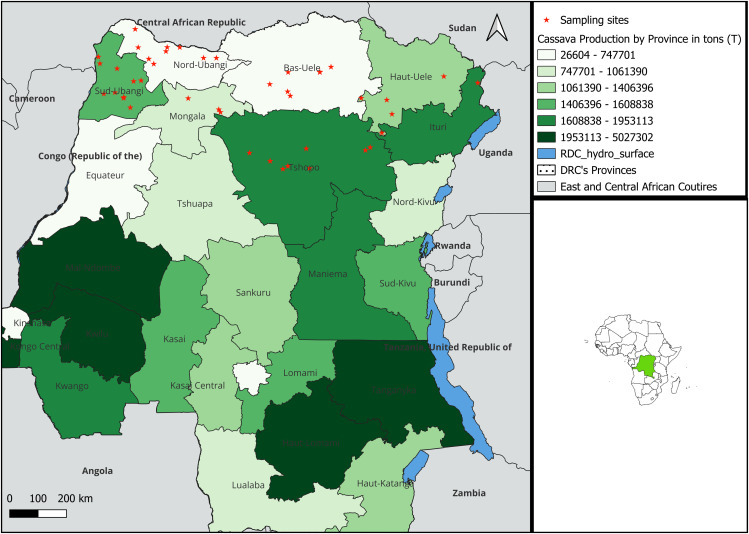
Importance of cassava production in tons (mt), whitefly sampling sites in the Democratic Republic of the Congo and neighbouring countries. All sites are numbered from 1 to 43 (East to West). This map was prepared by the first author using QGIS 3.36.3-Prizren software and GADM open-source shapefiles (https://gadm.org/maps.html).

Field sampling was performed in the DRC under the permission granted by Institut Facultaire des Sciences Agronomiques de Yangambi. The DRC is a tropical country with a maximum daily temperature that can exceed 35°C and a minimum temperature below 22°C, influenced by altitude in the northeast, east, southeast, plateau and mountain regions. Average annual precipitation is 1,612 mm and varies according to season. The length of the dry season follows a gradient from south to north, with southern regions experiencing a longer dry season than northern ones. This study was carried out between January and March 2022 in seven northern cassava production provinces of the DRC bordered by Uganda, South Sudan, the Central African Republic and the Republic of Congo. Adult whiteflies were collected from georeferenced cassava fields, 3–9 months after planting, using a mouth aspirator. Samples were immediately preserved in 2 ml Eppendorf tubes containing absolute alcohol (99%). Out of 116 potential sites, 43 were selected based on the criterion of having a minimum of 20 female whiteflies per site, with a minimum geographical distance of 10 km between neighbouring sites (Fig 1). The sampling map was drawn up from georeferenced data from each collection site using QGIS 3.36.3-Prizren software [31] and open license shapefiles downloaded online [32].

### DNA extraction

Identification of female whiteflies was based on morphological characteristics [33] at the Laboratoire de Phytopathologie et Biotechnologies végétales, Institut Facultaire des Sciences Agronomiques de Yangambi, under a stereomicroscope (Wild heerbrugg M1A; bontempsmicroscopie@skynet.be). All identified females were stored at −20°C. Genomic DNA from female whiteflies was extracted according to Ally *et al.* [18]. The extracted DNA was stored at −20°C until use.

### mtCOI PCR amplification, sequences analysis and species identification

The partial mitochondrial cytochrome oxidase I gene (mtCOI) was amplified using primers (2195Bt and C012/Bt-sh2) designed by Mugerwa *et al*. [20]. This was done to i) ensure only *B. tabaci s.l.* specimens were collected, as several morphologically similar whitefly species can be found in sympatry and could be misidentified at the adult stage, and ii) differentiate the cryptic species of the *B. tabaci s.l.* species complex. The PCR mix was prepared in 96-well plates, with a total volume of 25 μl, which contained 2 μl of DNA, 12.5 μl of 2x Type-it master mix (Qiagen; France); 8 μl of HPLC water and 2.5 μl of primer (forward and reverse) at 100 pmol/µl. The PCR program consisted of an initial denaturation at 95°C for 15 min, followed by 40 cycles of denaturation at 95°C for 30 s, hybridization at 52°C for 30 s, elongation at 72°C for 1 min and a final elongation at 72°C for 10 min. Amplified products were visualised using the QI Axel Advanced analyser (QI Axel ScreenGel® software; Qiagen). The dried amplified products were sequenced by Macrogen Europe. Generated sequences were manually edited and analysed under Geneious Rv10.2.6 [34], then compared with GenBank sequences using the BLAST algorithm. Sequence haplotypes were generated by DnaSP v6.12.03 [35]. The unique haplotypes were aligned with reference sequences from GenBank using ClustalW [36] in Geneious® 10.2.6. The Jmodeltest 2.1.10 software [37] was used to determine the optimal model for phylogenetic tree construction, and the tree was made using Mr Bayes [38] with the GTR + I + G substitution model [38], run with 1,000,000 iterations of MCMC (the first 100,000 iterations were rejected), and sample trees were made after every 200 iterations.

### Microsatellite genotyping and analysis

Following mtCOI analysis, only *B. tabaci s.l.* samples were selected for genotyping using 12 microsatellite marker pairs in three mixes. Full details of the multiplex preparation and PCR conditions are supplied in S1 Table. Microsatellite PCR products were diluted at 1/20 for the first two mixes and at 1/120 for mix 3. Subsequently, 2 μl of these dilutions were mixed with 10.7 μl of Hi-Di-formamide (Applied Biosystems) and 0.3 μl of size marker (GeneScan 500Liz) and denatured at 95 ºC for 5 min. Fragments were chilled on ice for 5 min before analysis on an Automated 3500xL Genetic Analyzer (Applied Biosystems). The fluorescent genotypic data were visualised using Geneious® 10.2.6 and each genotype was scored per sample.

For the structure analysis, *B. tabaci s.l.* individuals having more than 45% missing allele data were excluded (n = 5). Genepop 4.7.5 online software [39] and Genetix 4.05.2 [40] were used to calculate expected heterozygosity (He), expected heterozygosity calculated without bias (Hn. b), observed heterozygosity (Ho), the mean number of alleles per population and excess of heterozygotes (FIS) (Table 1). The genetic distance and geographic distance matrices were generated under Genepop 4.7.5.

**Table 1:** Population genetic diversity indices for *B. tabaci* SSA1 SG1 U SG2 sampled from Northern Democratic Republic of the Congo per site on cassava. The genetic indices for the two other *B. tabaci* species were not calculated due to too low number per site (Num site: Number of sites, Nbr Inds: number of individuals, Hexp: expected heterozygosity; Hnb: heterozygosity calculated without biases, Hobs: observed heterozygosity, F_IS_: is a measure of deviation from panmixia at local scales; P-value from Hardy-Weinberg equilibrium test: *, P < 0.05; **, P < 0.01; ***, P < 0.001). The population genetic diversity indices were calculated considering a minimum number of individuals of n > 5 per field, if this number was not reached a “– “was noted.

Num site	Nbr Inds	Mean alleles	Hexp.	H n.b.	Hobs.	F_is_
1	36	6.8333	0.4658 ± 0.2822	0.4726 ± 0.2863	0.3924 ± 0.2568	0.1653 ***
2	30	6.2500	0.4907 ± 0.2688	0.4992 ± 0.2733	0.3475 ± 0.2436	0.2979 ***
3	31	6.7500	0.5018 ± 0.2938	0.5108 ± 0.2988	0.3632 ± 0.2733	0.2724***
4	35	7.8333	0.4875 ± 0.2903	0.4948 ± 0.2945	0.3867 ± 0.2501	0.2125***
5	28	6.6667	0.4855 ± 0.2699	0.4946 ± 0.2749	0.3915 ± 0.2641	0.2057***
6	31	7.2500	0.5055 ± 0.2737	0.5141 ± 0.2782	0.4007 ± 0.2532	0.2181***
7	13	4.6667	0.4754 ± 0.2635	0.4952 ± 0.2742	0.3803 ± 0.2437	0.2352***
8	3	2.2500	0.3461 ± 0.2562	0.4194 ± 0.3083	0.4583 ± 0.3562	–
9	36	6.1667	0.4605 ± 0.2852	0.4670 ± 0.2892	0.3811 ± 0.2594	0.1868***
10	8	3.9167	0.4000 ± 0.3042	0.4272 ± 0.3242	0.3403 ± 0.2723	0.2190***
11	36	6.0833	0.4631 ± 0.2867	0.4697 ± 0.2907	0.3762 ± 0.2544	0.2007***
12	14	5.3333	0.5052 ± 0.3048	0.5250 ± 0.3167	0.3168 ± 0.2607	0.4027***
13	19	5.0833	0.5129 ± 0.2771	0.5269 ± 0.2847	0.3764 ± 0.2285	0.2906***
14	34	6.2500	0.4769 ± 0.3062	0.4850 ± 0.3113	0.3900 ± 0.2997	0.1748***
15	10	4.3333	0.4435 ± 0.3006	0.4673 ± 0.3164	0.3037 ± 0.2544	0.3608***
16	8	3.5833	0.4397 ± 0.3059	0.4723 ± 0.3287	0.4311 ± 0.3649	0.0701***
18	3	2.2500	0.3883 ± 0.2649	0.4496 ± 0.3039	0.2639 ± 0.3707	–
19	27	6.0000	0.4350 ± 0.3002	0.4437 ± 0.3055	0.3373 ± 0.3027	0.2192***
20	6	3.5000	0.4164 ± 0.2997	0.4586 ± 0.3267	0.3917 ± 0.2910	0.1693***
21	15	4.5000	0.4104 ± 0.2925	0.4249 ± 0.3029	0.3581 ± 0.2468	0.1619***
22	35	6.5000	0.4399 ± 0.2922	0.4465 ± 0.2964	0.3549 ± 0.2310	0.2053***
23	6	3.1667	0.4190 ± 0.3137	0.4587 ± 0.3420	0.2917 ± 0.2671	0.3713***
24	7	3.1667	0.3872 ± 0.2977	0.4180 ± 0.3210	0.3393 ± 0.2925	0.1939**
25	33	6.5833	0.4848 ± 0.2817	0.4929 ± 0.2863	0.3510 ± 0.2008	0.2825***
26	35	6.2500	0.4912 ± 0.2345	0.4993 ± 0.2378	0.3118 ± 0.1746	0.3570***
27	4	2.5833	0.4057 ± 0.2871	0.4687 ± 0.3309	0.3264 ± 0.2786	–
28	35	5.2500	0.4759 ± 0.2203	0.4835 ± 0.2234	0.2918 ± 0.1928	0.3800***
29	18	4.7500	0.4996 ± 0.2856	0.5191 ± 0.2990	0.3582 ± 0.2338	0.2725***
30	35	7.3333	0.5002 ± 0.2947	0.5090 ± 0.3003	0.3605 ± 0.2326	0.2459***
31	13	4.2500	0.4457 ± 0.2555	0.4692 ± 0.2699	0.4151 ± 0.2632	0.1036***
32	27	4.6667	0.4447 ± 0.2405	0.4536 ± 0.2449	0.2613 ± 0.1904	0.4133***
33	19	3.8333	0.3982 ± 0.2717	0.4100 ± 0.2793	0.2893 ± 0.2355	0.2813***
34	3	2.2500	0.4132 ± 0.2577	0.5028 ± 0.3164	0.4167 ± 0.3794	–
35	5	2.6667	0.3550 ± 0.2845	0.4037 ± 0.3228	0.2500 ± 0.2714	0.3651***
36	11	3.9167	0.4373 ± 0.2702	0.4609 ± 0.2832	0.3535 ± 0.2628	0.2496***
37	33	6.2500	0.4757 ± 0.2793	0.4847 ± 0.2846	0.3499 ± 0.2461	0.2333***
38	34	6.9167	0.4763 ± 0.2829	0.4847 ± 0.2878	0.3542 ± 0.2360	0.2401***
39	13	4.9167	0.4786 ± 0.3030	0.5012 ± 0.3144	0.4087 ± 0.3080	0.1510***
40	9	3.6667	0.4589 ± 0.2870	0.4916 ± 0.3045	0.2801 ± 0.2388	0.4082***
41	13	5.6667	0.5279 ± 0.2732	0.5507 ± 0.2851	0.3317 ± 0.2175	0.3979***
42	25	5.9167	0.5251 ± 0.2952	0.5372 ± 0.3017	0.3181 ± 0.2007	0.3929***
43	15	4.6667	0.4576 ± 0.2509	0.4765 ± 0.2600	0.3913 ± 0.2590	0.1549***

STRUCTURE v.2.3.4 was used to assign whiteflies individually to an unknown genetic population based on posterior probabilities obtained from multi-locus genotypic data [41]. The software was run 10 times with 10^5^ initial iterations as burn-in and then for 10^6^ MCMC iterations with K ranging from 1 to 20. The results were exported to the web-based software STRUCTURESELECTOR for post-STRUCTURE analysis [42]. The best cluster (K) was determined following Evanno [43], and graphical representations of the results were generated by integrating the CLUMPAK program under STRUCTURESELECTOR [42,44]. Mean genetic cluster assignments for each of the seven provinces were derived from the site-level data. Subsequently, a Discriminant Analysis of Principal Components (DAPC) was performed in R version 4.4.0 [45] using the adegenet package with microsatellite data from SSA1 and SSA3 populations.

## Results

### *Bemisia tabaci* species and its distribution

**Fig 2:**
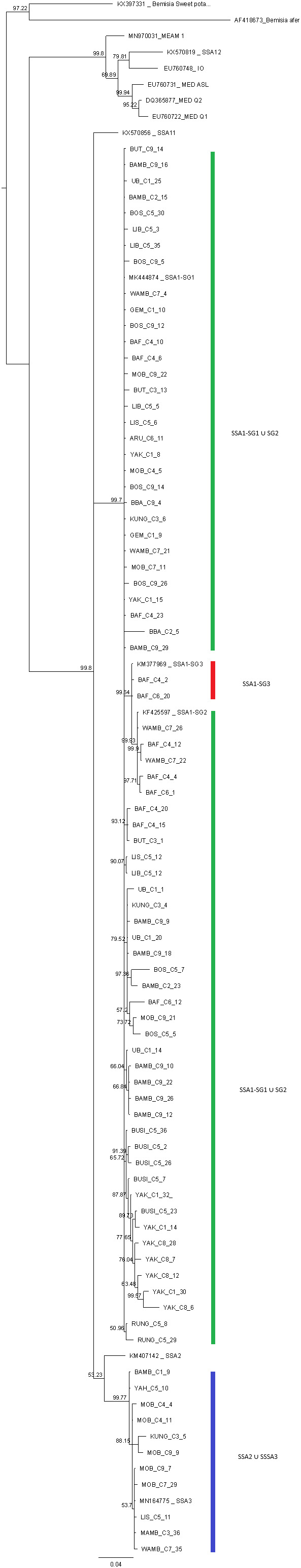
Phylogenetic tree generated by Mr Bayes, using 83 mtCOI haplotype sequences of 704 bp from adult *B. tabaci s.l.* of the DRC and 14 reference sequences from GenBank.

In this study, mtCOI fragments (705 bp) were successfully amplified and sequenced from 1,024 female whiteflies. One hundred seventy-one individuals were excluded, they were identified as *Bemisia afer* (n = 109) or unreadable sequences (n = 12). Three *B. tabaci s.l.* species were found after analysis of 903 whiteflies’ partial mtCOI sequences: *B. tabaci* SSA1-SG1 ∪ SG2 (n = 857: 94.91%), *B. tabaci* SSA2 ∪ SSA3 (n = 42: 4.65%), and *B. tabaci* SSA1-SG3 (n = 4: 0.4%). *B. tabaci* SSA1-SG1 ∪ SG2 was the most abundant and found in all seven surveyed provinces, while *B. tabaci* SSA2 ∪ SSA3 was present in the Nord-Ubangi provinces (n = 24), followed by Sud-Ubangi (n = 7); Bas-Uélé (n = 5); Mongala (n = 3); Tshopo (n = 2); Haut-Uélé (n = 2) and Ituri (n = 1) (Table 2). The two subgroups of *B. tabaci* SSA1-SG1 ∪ SG2 were found: -SG1 (n = 810), and -SG2 (n = 47). Sequence analysis revealed 83 different unique haplotypes (submitted in GenBank: PQ053239-PQ053321, Fig 2), with 71 identified as *B. tabaci* SSA1-SG1 ∪ SG2 (85.54% of diversity overall *B. tabaci* SSA1-SG1 ∪ SG2 samples), 2 identified as SSA1-SG3 (2.41%) and 10 as *B. tabaci* SSA2 ∪ SSA3 (12.05% of diversity overall *B. tabaci* SSA2 ∪ SSA3 samples). Two dominant haplotypes were found across species (SSA2 ∪ SSA3_MOB_C9_7_ = 76.19% (32/42) for *B. tabaci* SSA2 ∪ SSA3; SSA1_YAK_C1_15_ = 69.58% (597/858) for *B. tabaci* SSA1-SG1 ∪ SG2). The dominant *B. tabaci* SSA1-SG1 ∪ SG2 haplotype (YAK_C1_15, n = 597) was found in all the 42 sites sampled, and was 100% identical with GenBank sequences MK444874, MZ331390, and MT542017 found in Uganda [46], Kenya [47], and Zambia [48], respectively (Table 2). Only four individuals were identified as *B. tabaci* SSA1-SG3, all collected from the Ituri and Tshopo provinces. The dominant *B. tabaci* SSA2 ∪ SSA3 haplotype (MOB_C9_7) was widely distributed across the 15 sampled sites in the western provinces and showed 100% sequence identity with MN164775 [28] and KM377923 [49] previously reported from Nigeria.

**Table 2:** Distribution of the two dominant haplotypes of SSA2 ∪ SSA3 (MOB_C9_7) and SSA1-SG1 ∪ SG2 (YAK_C1_15) sampled from cassava plants in the northern Democratic Republic of the Congo, organised by site.

Provinces	Site number	Number of Individuals	Haplotype (Yak-C1-15)	Haplotype (MOB_C9_7)
Ituri	1	36	35	–
	2	30	20	–
Haut-Uélé	3	31	31	–
	4	35	29	1
	5	28	24	–
	6	31	25	–
Bas-Uélé	7	13	6	–
	8	3	3	2
	9	36	26	--
	10	8	7	–
	11	36	14	–
	12	14	6	–
	13	19	16	–
Tshopo	14	34	15	–
	15	10	4	–
	16	8	4	1
	18	3	3	–
	19	27	14	–
	20	6	3	–
Mongala	21	15	14	–
	22	35	30	–
	23	6	5	–
	24	7	5	2
Nord-Ubangi	25	33	26	–
	26	35	20	1
	27	4	2	2
	28	35	9	–
	29	18	16	8
	30	35	32	–
	31	13	8	5
	32	27	13	2
	33	19	7	2
Sud-Ubangi	34	3	3	1
	35	5	4	–
	36	11	9	–
	37	33	23	–
	38	34	31	–
	39	13	8	1
	40	9	6	2
	41	13	9	1
	42	25	19	1
	43	15	13	–

### Population diversity and structuring

**Fig 3:**
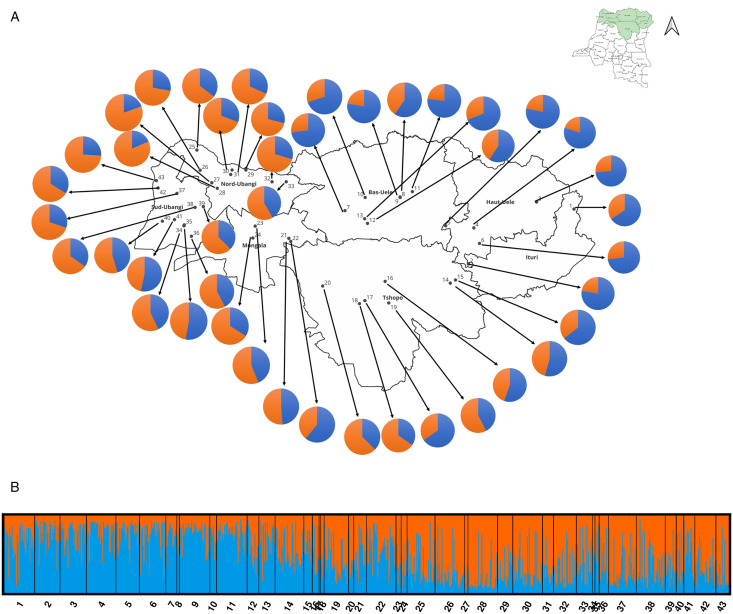
Bayesian clustering and geographic distribution of the genetic clusters of *Bemisia tabaci*  SSA1 populations in DRC. A. Distribution of the two genetic clusters of SSA1-SG1 U SG2 according to their site (sites 1-43). This map was prepared by the first author using QGIS 3.36.3-Prizren software and GADM open-source shapefiles (https://gadm.org/maps.html). B. Bayesian Structure bar plots based on 12 microsatellite loci for *B. tabaci* SSA1 SG1 U SG2 from Northern DRC (K = 2, N = 856).

**Fig 4:**
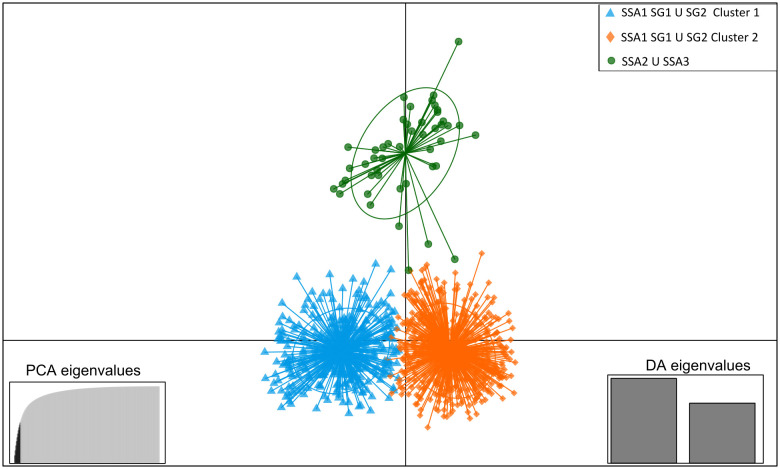
Discriminant Analysis of Principal Components (DAPC) performed on microsatellite data of *B. tabaci s.l.* species sampled using the adegenet package in R.4.4.0. Dots of different colours indicate individuals from the two different genetic clusters of *B. tabaci* SSA1 SG1 U SG2 (orange and blue) and of *B. tabaci* SSA2 U SSA3 (dark green). PCA eigenvalues and discriminant factors retained are indicated.

Population genetic diversity and structure analyses were performed on 856 *B. tabaci s.l.* samples. Population genetic diversity indices were only performed on *B. tabaci* SSA1 SG1 U SG2, which had enough individuals per site. Moderate heterozygosity levels were observed among populations for *B. tabaci* SSA1 SG1 U SG2 (in average over all populations: 0.3522 ± 0.0479, Table 1). The Fis values ranged from 0.2569 ± 0.0914, with significant departures from Hardy–Weinberg equilibrium observed for all populations sampled (Table 1). We observed a highly significant correlation between genetic (Fst) and geographical distances (Mantel’s test, P-value < 0.0001, S3 Fig) between populations (sites) of *B. tabaci* SSA1 SG1 U SG2 (this Mantel test was not performed on *B. tabaci* SG3 or SSA2 ∪ SSA3 due to low sample sizes). Bayesian genetic clustering analyses (i) well separated *B. tabaci* SSA2 U SSA3 and SSA1 SG1 U SG2 species (data not shown), and (ii) showed that *B. tabaci* SSA1 SG1 U SG2 from northern DRC were further subdivided into two genetic clusters (Best K population S4 Fig). The *B. tabaci* SSA1 SG1 U SG2 individuals assigned to these two genetic clusters were geographically distributed across the different provinces. One cluster was mainly observed in the four eastern provinces (Sites 1–22, cluster/blue pop) with a minority observed in the tree western provinces (Sites 23–43, blue/orange clusters, Fig 3A and B), as also illustrated by the average assignations per province (S2 Fig). The *B. tabaci* SSA1 SG1 U SG2 blue cluster (n = 443) had more individuals than the orange cluster (n = 413). The same pattern was observed for the distribution of the dominant *B. tabaci* SSA1 SG1 U SG2 (YAK_C1_15) and SSA2 U SSA3 (MOB_C9_7) haplotypes (Table 2). Different levels of hybridization between sites from each genetic cluster can be observed (Fig 3B) indicating a signal of population movement between sites. No hybridization was observed between *B. tabaci* SSA1 SG1 U SG2 and *B. tabaci* SSA2 U SSA3 populations, with high genetic assignment by Bayesian analysis (99.44%) of all individuals for each species (data not shown); DAPC analysis (Fig 4), confirmed that they belong to two different species.

## Discussion

*Bemisia tabaci* SSA1-SG1 U SG2 is dominant on cassava and is structured in two genetic clusters in the northern Democratic Republic of the Congo. Several cassava-colonising species have been identified in the eastern and central parts of sub-Saharan African countries [18,25,28]. Unlike these countries, our study reported only three species of cassava in northern DRC: *B. tabaci* SSA1-SG1 U SG2, *B. tabaci* SSA1-SG3, and *B. tabaci* SSA2 U SSA3, despite a very broad survey conducted over a 1339 km transect (Fig 2){Allsopp, 2010 #14129}{Allstadt, 2009 #14755}. *B. tabaci* SSA1-SG1 U SG2 was identified as the main species and was found in all fields in the provinces surveyed during the sampling period. Numerous other studies have also reported its high prevalence on cassava, such as in Uganda [25,26], Western and Nyanza regions of Kenya [47], Tanzania [18], Zambia [48], Central African Republic [23] and Eastern DRC [29], in contrast to Southern Sudan [50] and Nigeria [28] where it was less abundant on cassava. Superabundant populations observed in Uganda and Tanzania over the past three decades have highlighted their role in the spread of cassava mosaic disease and cassava brown streak disease in the Great Lakes region [14,30]. Within this species, two genetic clusters have been identified that can hybridize, with one cluster dominant in the higher-altitude regions of the eastern and northern parts of the country, following an altitudinal gradient (mean altitude from Nord-Ubangi to Ituri: 394–1,273 m). This highland genetic cluster almost exclusively carries the prevalent haplotype YAK_C1_15 (100% identity with most Ugandan haplotypes such as MK444874, MK444874, MK444874) found in all provinces. We hypothesize that it may have originated from Uganda, where climatic conditions and agrosystems are quite similar, and this country is also considered to be the centre of origin of the sub-Saharan *B. tabaci* species complex [20,46]. We are still able to detect traces of two genetic clusters in the sampled populations with a decreasing proportion of the highland genotypes in lowland sites (with significant isolation by distance, S3 Fig). It is rare to find evidence of population structuring by host plant or geography within a polyphagous species [51]. Population structure within the same or related species may be related to their life history traits [52]. The structure of *B. tabaci* SSA1-SG1 U SG2 observed in these two genetic clusters in the DRC could suggest that we are observing a quite recent invasion of one genetic cluster by hybridization with local lowland populations of *B. tabaci* SSA1-SG1 U SG2. This invasion process, through hybridization with local populations, may confer beneficial traits that trigger this process. Indeed, some traits such as better fitness or insecticide resistance in a population within a species, are traits that can promote invasion [53]. All of these traits acquired from gene flow between adapted and invasive local populations in polyphagous insects [54] are referred to as the invasion syndrome [55]. A species can invade a region previously occupied by other species of the same genus or by other genotypes of the same species [56] and hybridize with them [57]. For example, MEAM1 (Middle East-Asia Minor) invaded Réunion Island since 1997. It is currently hybridising with the native IO (Indian Ocean) species, with a possible introgression of Pyretrinoid susceptibility into the invader, which is known to be resistant [17,58]. This invasion could also result from recent changes in key environmental variables affecting the distribution of *B. tabaci s.l.* such as land cover, precipitation and annual temperature range [59]. These changes drive interactions between invasive and native species, triggering introgressive invasive hybridization, and potentially causing the genetic extinction or displacement of many local species [60].

*B tabaci s.l.* is one of the key factors in the spread of viruses that cause CMD and CBSD in the DRC [8,29,61,62]. In the DRC, CBSD was first reported in the eastern part of the country [30], and is now spreading towards the western region [9], following the same invasion pattern revealed by the SSA1 genetic clusters. However, the rate of transmission of CBSD viruses by *B. tabaci s.l.* remains very low [12] and may depend on the species, hence the need to study the transmission efficiency of these viruses according to the different species of the *B. tabaci s.l.* species. The spread of CBSD in the DRC could be partly the result of the invasion by hybridization observed and represents a serious threat for West Africa where cassava production for industry is more developed, such as in Nigeria, the world’s largest producer of cassava [63].

The *B. tabaci* SSA2 U SSA3 species, although present in the DRC, has not been found as abundant on cassava as in Nigeria [28,49]. Its wide distribution remains in the Nord-Ubangi and Sud-Ubangi provinces bordering the Central African Republic, where it has also been found on cassava as the second most abundant species [23]. This study reported a low prevalence of *B. tabaci* SSA2 U SSA3 in eastern provinces bordering Uganda where it was recently identified for the first time [26]. Our results suggest that this species may be poorly adapted to high altitudes. *B. tabaci* SSA2 U SSA3 may be better adapted to sub-humid regions and would be less involved in the spread of CMD [28]. In the north-western provinces, the incidence of CMD is still moderate and its severity remains below level 3 [8], and no CBSD has been confirmed in this part of the country.

## Conclusion

Three whitefly species were found on cassava, *B. tabaci* SSA1 SG1 U SG2, *B. tabaci* SSA1 SG3 and *B. tabaci* SSA2 U SSA3, and a genetic structuring into two clusters of the *B. tabaci* SSA1 SG1 U SG2. Hybridization between the two genetic clusters from the most abundant *B. tabaci* SSA1 SG1 U SG2 populations was detected with a significant geographical structuring. This hybridization being one of the major features of the invasion syndrome, may enhance the adaptive potential of invasive populations from high eastern altitudes to low western altitudes (in similar regions or new environments) and is a signal for population movements, from the areas where outbreaks of CBSD have been reported to potentially CBSD-free areas. A better knowledge of the invasion syndrome within *B. tabaci* SSA1 populations is essential for effective management measures of this vector, to restrict the spread of CBSD in West Africa.

## Supporting information

S1 Table Microsatellite genotyping loci and multiplex PCR conditions. (DOCX)

S2 Fig Mean assignments of SSA1 individuals in two genetic clusters by province. (TIF)

S3 Fig Plot showing isolation by distance between genetic distance and geographic distance of SSA1 populations. (TIF)

S4 Fig Delta K of SSA1 population from structure harvester under Structure Selector. (PNG)

S5 File Inclusivity in global research questionnaire. (DOCX)
