# Supplementary material for: Molecular evidence of recent hybridization between eastern and western populations of a whitefly species on cassava in the Democratic Republic of the Congo: A potential threat to the spread of cassava brown streak disease
Source: PLoS One. 2026 Mar 31;21(3):e0338200. doi: 10.1371/journal.pone.0338200 (PMC13037998; doi:10.1371/journal.pone.0338200)

**S1 Table. Microsatellite genotyping loci and multiplex PCR conditions.**

| **Mixes** | **LN** | **Primer sequence** | **Motif** | **FL** | **%MS** | **Range** | **PCR conditions** | | |  |
| --- | --- | --- | --- | --- | --- | --- | --- | --- | --- | --- |
| Mix 1 | MS145 | F: CCTACCCATGAGAGCGGTAA | (AC)9 | PET | 4.2 | 124-234 |  |  |  |  |
|  |  | R: TCAACAAACGCGTTCTTCAC |  |  |  |  |  |  |  |  |
|  | P59 | F: CGGCGTTTCTCGTTTTCTT | (T)44(G)18 | 6-FAM | 2.3 | 148-220 |  |  |  |  |
|  |  | R: TTTGCCAACTGAAGCACATCAATCA |  |  |  |  | 95°C |  | 15 min |  |
|  | P7 | F: AGGGTGTCAGGTCAGGTAGC | 8(GT) | VIC | 2 | 117-287 | 95°C | → | 30 sec |  |
|  |  | R: TTTGCGTAATAGAAAA |  |  |  |  | 55°C | → | 1 min 30 sec | 40 cycles |
|  | WF2H06 | F: TATTCGCCAATCGATTCCTT | (TTTG)11 | NED | 4.4 | 102-214 | 72°C | → | 1 min |  |
|  |  | R: CGGCGGAAATTTCGATAAA |  |  |  |  | 60°C |  | 15 min |  |
| Mix 2 | P62 | F: CTTCCTTAGCACGGCAGAAT | (GT)8 | 6-FAM | 2.2 | 126-288 |  |  |  |  |
|  |  | R: TTTGGCGCAATTTTTAGCGTCTGT |  |  |  |  |  |  |  |  |
|  | WF1D04 | F: GTTGTTAGGTTACAGGGTTTGTC | (CAAA)16 | VIC | 1.8 | 100-172 |  |  |  |  |
|  |  | R: GTCTTTACTTCTTTTCCTCCG |  |  |  |  |  |  |  |  |
|  | P5 | F: ATTAGCCTTGCTTGGGTCCT | (GT)8 | NED | 4.8 | 100-288 |  |  |  |  |
|  |  | R: TTTGCAAAAACAAAAGCATGTGTCAAA |  |  |  |  |  |  |  |  |
| Mix 3 | CIRSSA2 | F: ACAATGCATGTTGATTGTGAA | (AG)6 | VIC | 0.2 | 100-126 |  |  |  |  |
|  |  | R: TGAAAATGTCTACGGCCAGA |  |  |  |  |  |  |  |  |
|  | CIRSSA6 | F: CATATCGGTCATTATCCGCA | (TC)6 | VIC | 0.2 | 117-173 | 95°C |  | 15 min |  |
|  |  | R: CATCAGGCTGGAAAGACGAG |  |  |  |  | 95°C | → | 30 sec |  |
|  | CIRSSA7 | F: TGGCGATCCTCTTCTTGTTT | (TC)5 | PET | 0.2 | 122-152 | 56°C | → | 1 min 30 sec | 40 cycles |
|  |  | R: AAGAAGCAGCAGTTCATCCG |  |  |  |  | 72°C | → | 1 min |  |
|  | CIRSSA13 | F: AGTGCTGAAGGTCCACCGTA | (CT)6 | NED | 12.8 | 145-291 | 60°C |  | 15 min |  |
|  |  | R: GGGATTTCCAGGGGTTAAGA |  |  |  |  |  |  |  |  |
|  | CIRSSA41 | F: TGGGTGCATGGTTCTTACAG | (CT)6 | 6-FAM | 25 | 112-184 |  |  |  |  |
|  |  | R: TATCCGGTCGACAAACACAA |  |  |  |  |  |  |  |  |

Locus name (LN), source reference, primer sequence, microsatellite repeat motif, fluorochromes used for labelling primers

**S2 Fig. Mean assignments of SSA1 individuals in two genetic clusters by province.**

**S3 Fig. Plot showing isolation by distance between genetic distance and geographic distance of SSA1 populations in Km.**

**S4 Fig. Delta K of SSA1 population from structure harvester under Structure Selector.**


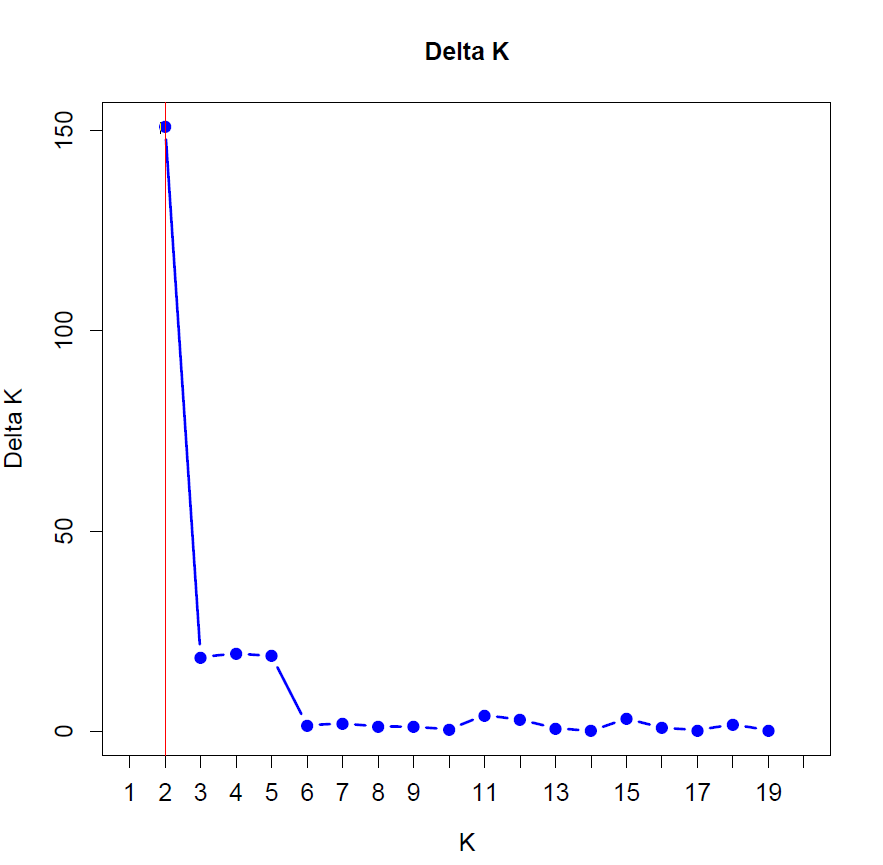

Supplement: S1 Table — (DOCX) [file pone.0338200.s001.docx]
