# Supplementary figures and images for: Molecular evidence of recent hybridization between eastern and western populations of a whitefly species on cassava in the Democratic Republic of the Congo: A potential threat to the spread of cassava brown streak disease
Source: PLoS One. 2026 Mar 31;21(3):e0338200. doi: 10.1371/journal.pone.0338200 (PMC13037998; doi:10.1371/journal.pone.0338200)

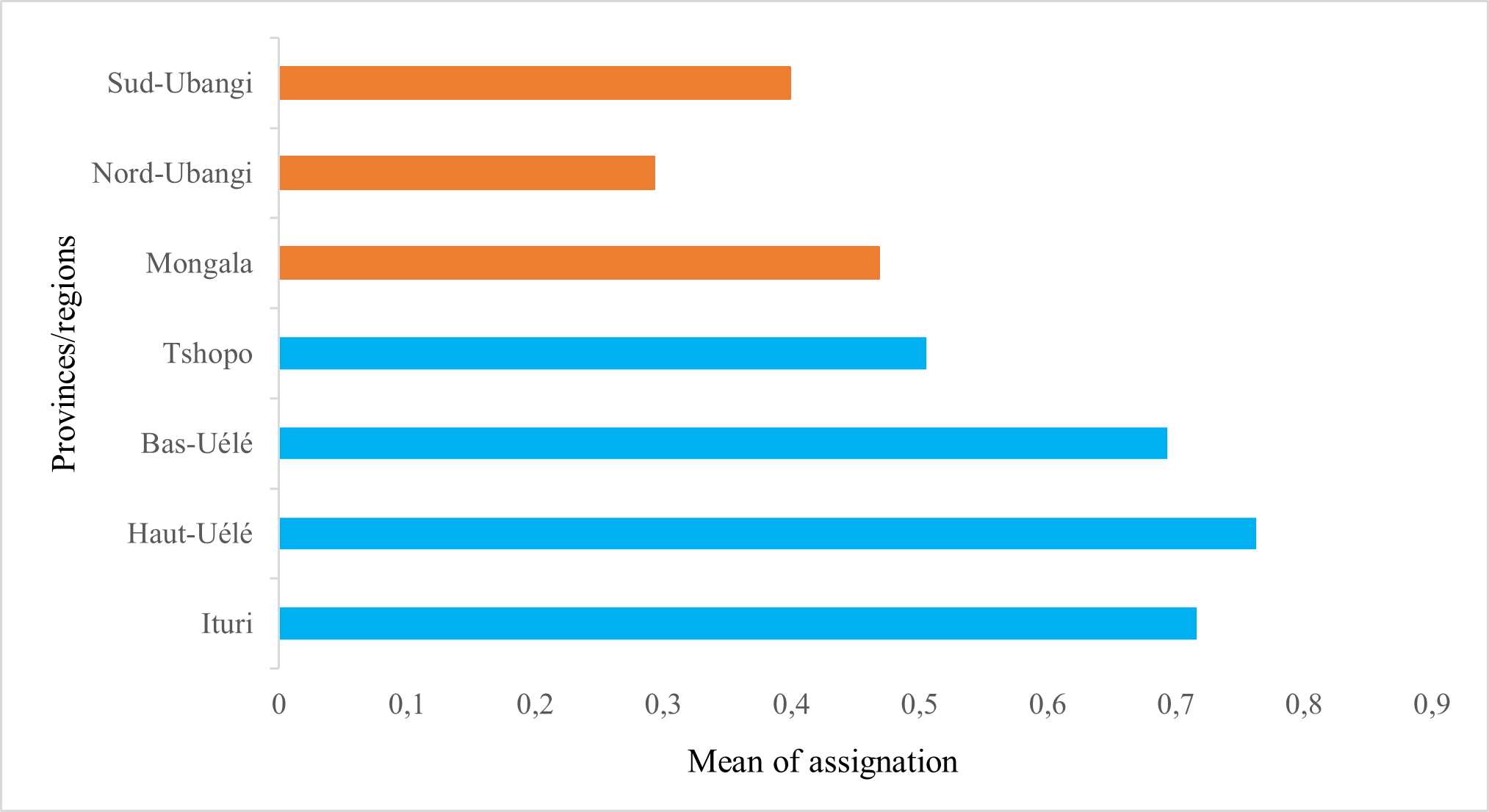

Supplement: S2 Fig — (TIF) [file pone.0338200.s002.tif]

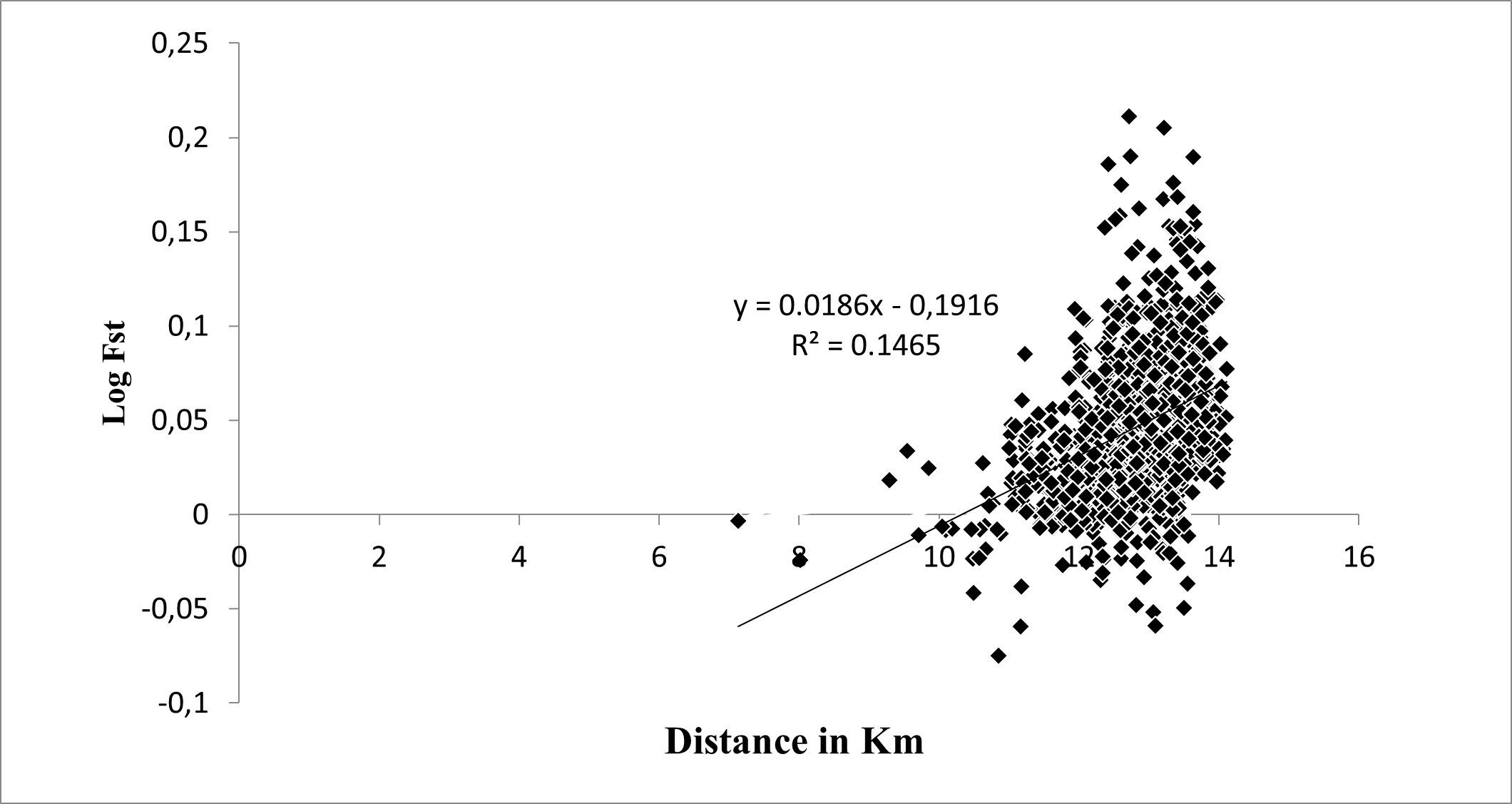

Supplement: S3 Fig — (TIF) [file pone.0338200.s003.tif]

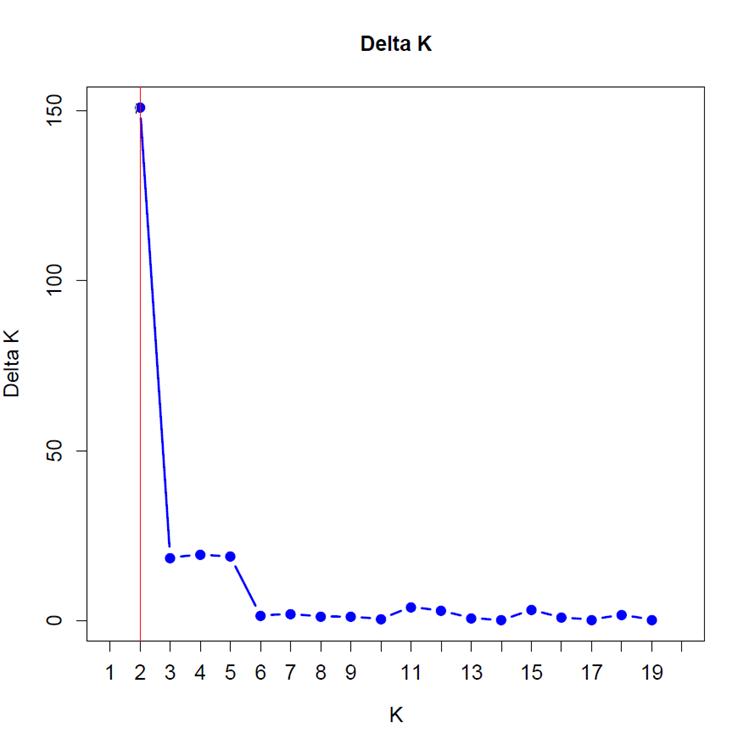

Supplement: S4 Fig — (PNG) [file pone.0338200.s004.png]
